# Repetitive transcranial magnetic stimulation over the supplementary motor area modifies breathing pattern in response to inspiratory loading in normal humans

**DOI:** 10.3389/fphys.2015.00273

**Published:** 2015-09-29

**Authors:** Marie-Cécile Nierat, Anna L. Hudson, Joël Chaskalovic, Thomas Similowski, Louis Laviolette

**Affiliations:** ^1^UMR S1158 Neurophysiologie Respiratoire Expérimentale et Clinique, Institut National de la Santé et de la Recherche Médicale, University Pierre et Marie CurieParis, France; ^2^Neuroscience Research Australia and University of New South WalesSydney, NSW, Australia; ^3^Institut Jean Le Rond D'Alembert, University Pierre et Marie CurieParis, France; ^4^Assistance Publique - Hôpitaux de Paris, Groupe Hospitalier Pitié-Salpêtrière Charles Foix, Service de Pneumologie et Réanimation MédicaleParis, France; ^5^Centre de Recherche de l'Institut de Cardiologie et de Pneumologie de QuébecQuébec, QC, Canada

**Keywords:** breathing pattern, cerebral cortex, control of breathing, dyspnea, inspiratory loading, transcranial magnetic stimulation

## Abstract

In awake humans, breathing depends on automatic brainstem pattern generators. It is also heavily influenced by cortical networks. For example, functional magnetic resonance imaging and electroencephalographic data show that the supplementary motor area becomes active when breathing is made difficult by inspiratory mechanical loads like resistances or threshold valves, which is associated with perceived respiratory discomfort. We hypothesized that manipulating the excitability of the supplementary motor area with repetitive transcranial magnetic stimulation would modify the breathing pattern response to an experimental inspiratory load and possibly respiratory discomfort. Seven subjects (three men, age 25 ± 4) were studied. Breathing pattern and respiratory discomfort during inspiratory loading were described before and after conditioning the supplementary motor area with repetitive stimulation, using an excitatory paradigm (5 Hz stimulation), an inhibitory paradigm, or sham stimulation. No significant change in breathing pattern during loading was observed after sham conditioning. Excitatory conditioning shortened inspiratory time (*p* = 0.001), decreased tidal volume (*p* = 0.016), and decreased ventilation (*p* = 0.003), as corroborated by an increased end-tidal expired carbon dioxide (*p* = 0.013). Inhibitory conditioning did not affect ventilation, but lengthened expiratory time (*p* = 0.031). Respiratory discomfort was mild under baseline conditions, and unchanged after conditioning of the supplementary motor area. This is the first study to show that repetitive transcranial magnetic stimulation conditioning of the cerebral cortex can alter breathing pattern. A 5 Hz conditioning protocol, known to enhance corticophrenic excitability, can reduce the amount of hyperventilation induced by inspiratory threshold loading. Further studies are needed to determine whether and under what circumstances rTMS can have an effect on dyspnoea.

## Introduction

In awake humans at rest, lung ventilation depends on the homeostatically regulated cyclical activity of brainstem pattern generators. Ventilation decreases during sleep, suggesting the existence of a “*wakefulness drive to breathe,*” of which best known manifestation is the fact that hyperventilation-induced hypocapnia induces apneas during sleep, but fails to do so during wakefulness (Fink, 1961b; Datta et al., 1991; Corfield et al., 1995). The neurophysiological determinants of the wakefulness drive include a cortical component (Fink, 1961a; Shea, 1996; Mehiri et al., 2006). Recently, we posited that the supplementary motor area (SMA) contributes to the wakefulness drive to breathe through its respiratory connections (Laviolette et al., 2013). Indeed, an inhibitory repetitive magnetic stimulation (rTMS) paradigm applied to the SMA during quiet natural breathing depressed the excitability of the corticospinal pathway to the diaphragm in healthy humans (Laviolette et al., 2013). The contribution of the SMA to the wakefulness drive to breathe was subsequently corroborated by the presence of premotor potentials, detected by electroencephalographic recordings, preceding inspiration in patients with defective automatic breathing who are characterized by the strict dependency of their ventilatory activity on wakefulness (Tremoureux et al., 2014). Premotor potentials originate, in part, in the SMA (Ball et al., 1999).

The SMA and other cortical areas that contribute to premotor potentials are also activated in response to inspiratory mechanical constraints (generally referred to as “inspiratory loads,” a generic term that designate any experimental device imposing an increased effort on the respiratory system, e.g., resistances, elastances, and threshold valves) in normal humans (Raux et al., 2007a,b, 2013). This cortical activation is hypothetized to constitute an adaptative reaction, although its exact pathway is not known. It occurs in conjunction with altered breathing pattern, usually hyperventilation (“overcompensation”; Freedman and Weinstein, 1965; Yanos et al., 1990). It is also accompagnied by breathing discomfort (Raux et al., 2007a, 2013).

We hypothesized that manipulating SMA-excitability with rTMS would modify the breathing pattern response to inspiratory loading, and possibly also the corresponding respiratory sensations. Because the effects of rTMS on motor behaviors are complex and influenced by many factors, we investigated the effects of both an excitatory protocol (5 Hz stimulation) and an inhibitory protocol (continuous theta burst stimulation, cTBS) on breathing pattern and respiratory discomfort during inspiratory threshold loading in healthy subjects. Specifically, we hypothesized that there would be greater load-induced hyperventilation following 5 Hz stimulation due to increased excitability of the corticophrenic pathways via SMA-M1 connections and conversely, that hyperventilation would be less following inhibition of these connections with cTBS stimulation. A sham stimulation paradigm was used as control.

## Materials and methods

### Subjects

After approval by the appropriate French ethics and regulatory authorities (*Comité de Protection des Personnes Ile-de-*France VI Pitié-Salpêtrière, Paris, France), seven healthy subjects (three men; age: 25 ± 4 years; height: 174.7 ± 11.7 cm; weight: 73.7 ± 15.0 kg; mean ± SD participated in the study. They had no history of respiratory or neuromuscular disease and were all right-handed. They had previously received rTMS, but had no previous experience with experimental inspiratory loads. Subjects received detailed information and gave written informed consent.

### Experimental design

Each subject participated in three sessions (excitatory rTMS, inhibitory rTMS, and sham stimulation; see below), with at least 3 days between any two sessions. A single-blind design was used and the order of the stimulation was randomized for each subject. Each session consisted of (1) a 15-min epoch of inspiratory threshold loading (ITL); (2) 15 min of unloaded free breathing to let the subject recover; (3) rTMS conditioning of the supplementary motor area (SMA) with excitatory, inhibitory or sham stimulation; (4) a second 15-min session of ITL. For ITL, a spring threshold inspiratory muscle trainer (7–41 cm H_2_O, Health Scan, NJ, USA) was attached to the inspiratory arm of the breathing circuit. Because maximal inspiratory pressure (*P*_I, max_) depends on gender, age, age, weight, and other factors (Hautmann et al., 2000), we did not use a fixed value for the inspiratory load but rather adapted it to represent 25% of *P*_I, max_ (range 20–35 cm H_2_O across subjects), in an attempt to maximize between-subjects homogeneity. Throughout the experiments, the subjects sat in a comfortable chair with the head supported, and were distracted from experimental cues by watching a movie projected on a television screen in front of them.

### Respiratory measurements

Subjects breathed through a mouthpiece connected in series with a heated pneumotachograph (3700 series, linearity range 0–160 L^*^min^−1^; Hans Rudolph, Kansas City, MO) and a two-way valve (Hans Rudolph 2600 medium, Kansas City, MO). The experimental apparatus had a resistance < 1 cm H_2_O^*^L^−1^^*^s^−1^ and its dead space was ~40 mL. Ventilatory airflow (V′) was measured by connecting the pneumotachograph to a linear differential pressure transducer (± 5 cm H_2_O, DP45-18, Validyne, Northridge, CA). Tidal volume (*V*_T_) was obtained by electrical integration of flow. Inspiratory time (*T*_I_), expiratory time (*T*_E_), and total time (*T*_T_), breathing frequency (*f*), mean inspiratory flow (*V*_T_/*T*_I_), and duty cycle (*T*_I_/*T*_T_) were obtained by offline signal analysis (Chart™ software, AD Instruments, Castle Hill, Australia).

Inspiratory airway opening pressure was measured by a differential pressure transducer (±100 cm H_2_O, DP15-34, Validyne, Northridge, CA) connected to a lateral port of the mouthpiece and peak negative values for each respiratory cycle were used in the analysis. End-tidal carbon dioxide tension (PETCO2) was measured at a lateral port of mouthpiece with an infrared CO_2_ analyser (Servomex 1505, Plaine Seine Saint-Denis, France). All respiratory signals were recorded through an analog-digital converter (Maclab 16S, Powerlab System, AD Instruments, Castle Hill, Australia; sampling rate 2000 Hz) and Chart™ software (Chart 5.0, AD Instruments, Castle Hill, Australia). The degree of “respiratory discomfort” was self-assessed by the subjects on a visual analog scale (VAS). The subjects were asked to set the cursor on a 10 cm horizontal line, between the descriptions “no respiratory discomfort” and “intolerable respiratory discomfort.”

### Electromyographic recordings

Electromyographic recordings (EMG) were obtained using pairs of surface electrodes (self-adhesive hydrogel, diameter 20 mm, Comepa, St-Denis, France) placed on cleaned and abraded skin. A reference electrode was placed on one acromion. Surface EMG of the right first dorsal interosseous (FDI) and right abductor hallucis (AH) was recorded with tendon-belly arrangements. The FDI recording was used to determine the intensity of rTMS for conditioning (see below). In addition, for safety, FDI recordings were monitored because an increase in FDI activity during rTMS suggests spread of the stimulus to M1, with a potential risk of seizure (Rossi et al., 2009; Lefaucheur et al., 2011). The AH recording was used to locate the SMA (Matsunaga et al., 2005). EMG signals were amplified (x 1000) and filtered (band-pass 10 Hz–1 kHz) using a 1902 signal conditioner (Cambridge Electronic Design Ltd., Cambridge, UK), digitized at 10 kHz using a CED Power 1401 MkII data acquisition interface (Cambridge Electronic Design Ltd., Cambridge, UK) and stored on a personal computer for offline analysis using Signal software (Signal 5.00, Cambridge Electronic Design Ltd., Cambridge, UK).

### Repetitive transcranial magnetic stimulation (rTMS)

#### Motor hot spots and thresholds

All stimuli were delivered via a figure-of-eight coil (loop diameter: 70 mm). For FDI, the coil was connected to a Magstim Bistim^2^ super-rapid stimulator (Magstim, Whitland, UK) and placed tangentially over the scalp with the handle pointing backwards with a 45° angle to the mid-sagittal plane (Brasil-Neto et al., 1992). For AH the coil was connected to a Magstim 200 stimulator (Magstim, Whitland, UK) and placed along the sagittal midline with the handle pointing to the right. The motor hot spots (M1 cortical area) for the relaxed FDI and AH (i.e., no pre-stimulus EMG activity observed online) were determined as the single-pulse TMS (spTMS) coil position that elicited the largest MEP from the target muscle. For both muscles, the active motor threshold (AMT), determined during muscle contraction (20% of maximum voluntary activity, monitored with EMG), corresponded to the stimulation intensity (% of maximum stimulator output) that elicited an MEP ≥ 200 μV for 5 out of 10 successive stimuli. Motor thresholds were determined at each visit.

#### SMA localization

The SMA was identified as being 1 cm anterior to the most anterior point from which an AH MEP could be elicited under active contraction conditions (20% of maximum voluntary activity), with a stimulation intensity of 120% of the AH AMT. This was determined by moving the stimulation coil anteriorly from the AH hotspot over the midsagittal line, in agreement with a previously established procedure (Matsunaga et al., 2005). To best ensure the precise localization of the stimulation site and its stability throughout the experiments, a neuronavigation system (eXimia 2.2.0, Nextim Ltd., Helsinki, Finland) was used. For each subject, an anatomical MRI was analysed retrospectively to validate the positioning of stimulation coil over the SMA (Picard and Strick, 2001), which was always confirmed. It should be noted that without resorting to combined rTMS-fMRI, it is never possible to be absolutely certain of the structures stimulated by rTMS; we nevertheless took state-of-the-art procedures to maximize the likelihood of an actual SMA conditioning.

#### SMA conditioning

The SMA was conditioned with the figure-of-eight coil connected to the biphasic Magstim Bistim^2^ super-rapid stimulator. Three protocols were used, as follows:

1. *Inhibitory theta-burst stimulation, cTBS.* Three 50 Hz pulses repeated every 200 ms (i.e., at 5 Hz) continuously for 40 s (for a total of 600 pulses) were delivered at 80% of the AMT of the FDI over the SMA (Di Lazzaro et al., 2005; Huang et al., 2005; Laviolette et al., 2013).
2. *Facilitatory 5 Hz stimulation.* Ten second 5-Hz trains, separated by 50 s inter-train intervals, repeated 10 times (for a total of 500 pulses) were delivered over the SMA at 110% of the AMT of the FDI. This facilitatory protocol was selected as it has been previously shown to increase the evoked diaphragmatic response to corticospinal input following SMA conditioning (Raux et al., 2010; Laviolette et al., 2013).
3. *Sham stimulation.* The figure of eight coil was positioned on the scalp in the same way as during the active stimulations, but it was not connected to the stimulator. A second coil connected to the stimulator but kept at least 5 cm above the scalp was used to reproduce the stimulation related noises. Subjects could not see the position of the coils. Of note, the sham stimulation procedure was chosen in such a way as to conform with current recommendations on rTMS stipulating that “*The ideal placebo rTMS should fulfill a number of criteria: (i) the position of the active and placebo coils over the scalp should be the same; (ii) the subjective somatic scalp sensation (due to activation of superficial nerves/muscles) and the acoustic artifacts during stimulation should also be the same for active and sham coils, but (iii) no physiological effect on the targeted cortical region should occur for the placebo stimulation*” (Lefaucheur et al., 2014). Our approach did fulfill all the above criteria except the “somatic scalp sensation” one, but during *post-hoc* debriefings our subjects consistently reported not having experienced such sensations during any of the stimulation sessions.

### Statistical analysis and data management

For a reference baseline measure (BL), the last 10 min only of the first ITL session were analyzed. The first 5 min were discarded from this analysis to eliminate the period in which, presumably, discovery and habituation to the load occurred. For analysis, the second ITL session (following SMA conditioning) was segmented into three 5′ epochs (i.e., POST1, POST2, POST3) to determine if there were time-dependent changes in breathing pattern and discomfort, and for comparison with changes in diaphragmatic MEPs following similar SMA conditioning (Raux et al., 2010; Laviolette et al., 2013).

For each data set, the normality of the distribution (Shapiro–Wilk test) and the equality of variance (Levene's test) were checked. Normal data are described as mean values ± SEM and non-normal data as median and interquartile range. Statistical analyses were performed using one-way repeated-measures analysis of variance (ANOVA), or Friedman non-parametric ANOVA for normal, or non-normal data, respectively. *Post-hoc* pairwise comparisons were conducted for significant *F*-values (ANOVA) or significant χ^2^-values (Friedman). Statistical significance was assumed if *P* < 0.05, taking into account Hochberg–Benjamini correction for multiplicity (Hochberg and Benjamini, 1990; Benjamini and Hochberg, 1995). All calculations were performed using Sigmaplot (Systat Software, San Jose, CA), except for the multiplicity correction that was performed with a custom Microsoft Excel® spread sheet (courtesy Manuel Weinkauf, http://www.marum.de/en/Manuel_Weinkauf.html).

## Results

### Tolerance and safety

None of the subjects reported discomfort or abnormal sensations during or after the rTMS conditioning of the SMA. FDI EMG did not suggest spread of the rTMS stimulus to the primary motor cortex that would have been indicative of a seizure risk.

### Motor thresholds

No change in the AMT of the FDI and AH were observed between visits.

### Breathing pattern

Median resting ventilation, measured during the five last minutes of the unloaded resting breathing period was 6.95 L.min^−1^ [IQR: 6.42–10.64]. As expected (Yanos et al., 1990), ITL was associated with increased ventilation from these resting values (Tables 1, 2).

**Table 1:** **Effect of 5 Hz excitatory rTMS conditioning of the supplementary motor area (SMA) during inspiratory threshold loading on breathing pattern**.

	**5 Hz excitatory conditioning**			
	**BL**	**P1**	**P2**	**P3**
*T*_I_	2.32 [2.17–2.60]	1.93 [1.75–4.24]	**1.89 [1.60–1.92]**	**1.73 [1.61–1.97]**
			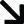	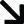
*T*_E_	4.76 (0.77)	5.50 (0.90)	6.16 (1.06)	5.76 (0.81)
*T*_T_	7.59 (0.86)	8.05 (0.79)	8.45 (0.87)	7.95 (0.65)
*T*_I_/*T*_T_	38.51 (4.88)	32.93 (7.37)	**30.22 (6.90)**	**29.29 (6.07)**
			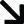	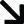
*f*	8.73 (1.03)	8.06 (0.99)	7.63 (1.10)	7.86 (0.88)
*V*_T_	1.61 [1.15–2.18]	1.70 [0.74–2.25]	**1.36 [0.79–2.01]**	1.51 [0.89–2.17]
			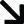	
*V*_T_/*T*_I_	0.49 [0.28–0.92]	0.66 [0.46–1.18]	0.58 [0.55–1.10]	0.71 [0.57–1.13]
$V_{\text{E}}^{\prime }$	15.75 (3.14)	**12.37 (2.71)**	**10.68 (2.11)**	**12.15 (2.97)**
		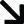	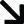	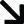
PetCO2	35.20 [30.94–37.50]	38.34 [36.06–42.37]	**38.41 [36.84–41.42]**	36.06 [33.39–41.37]
			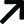	

BL, baseline; POST1, mean (SEM) or median [interquartile] over the first 5 min post-conditioning; POST2, over the second 5 min epoch; POST3, over the third 5 min epoch. T_I_, inspiratory time (s); T_E_, expiratory time (s); T_T_, total cycle time (s); T_I_/T_T_, duty cycle (%); V_T_, tidal volume (L); V_T_/T_I_, mean inspiratory flow (L.min^−1^); $V_{E}^{\prime }$, ventilation (L.min^−1^); PETCO2, end-tidal expired carbon dioxide (mmHg). Significant differences from BL are shown in bold and arrows indicate the direction of effect (p < 0.05).

**Table 2:** **Effect of cTBS inhibitory rTMS conditioning of the supplementary motor area (SMA) during inspiratory threshold loading on breathing pattern**.

	**cTBS inhibitory conditioning**			
	**BL**	**P1**	**P2**	**P3**
*T*_I_	2.22 (0.22)	2.08 (0.24)	2.12 (0.26)	1.95 (0.20)
*T*_E_	4.02 (0.48)	4.45 (0.65)	**5.69 (0.53)**	4.76 (0.77)
			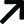	
*T*_T_	6.79 [4.94–7.36]	5.83 [5.10–8.54]	6.47 [5.84–9.11]	6.29 [5.52–9.62]
*T*_I_/*T*_T_	37.45 (3.62)	34.54 (4.03)	**32.76 (3.97)**	**30.99 (3.44)**
			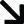	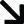
*f*	10.33 (1.11)	10.03 (1.37)	9.84 (1.81)	10.31 (2.24)
*V*_T_	1.58 (0.34)	1.68 (0.33)	1.57 (0.29)	1.66 (0.30)
*V*_T_/*T*_I_	0.65 [0.37–0.89]	0.43 [0.31–1.63]	0.48 [0.29–1.75]	0.53 [0.30–1.87]
$V_{\text{E}}^{\prime }$	14.43 (2.52)	15.30 (3.15)	13.57 (2.39)	14.44 (2.46)
PetCO2	34.37 [31.88–38.36]	35.82 [32.14–36.90]	34.02 [29.53–35.39]	34.05 [31.46–34.58]

Mean (SEM) or median [interquartile] data and abbreviations as for Table 1. Values in bold and arrows indicate significant differences from BL (p < 0.05).

**Figure 1:**
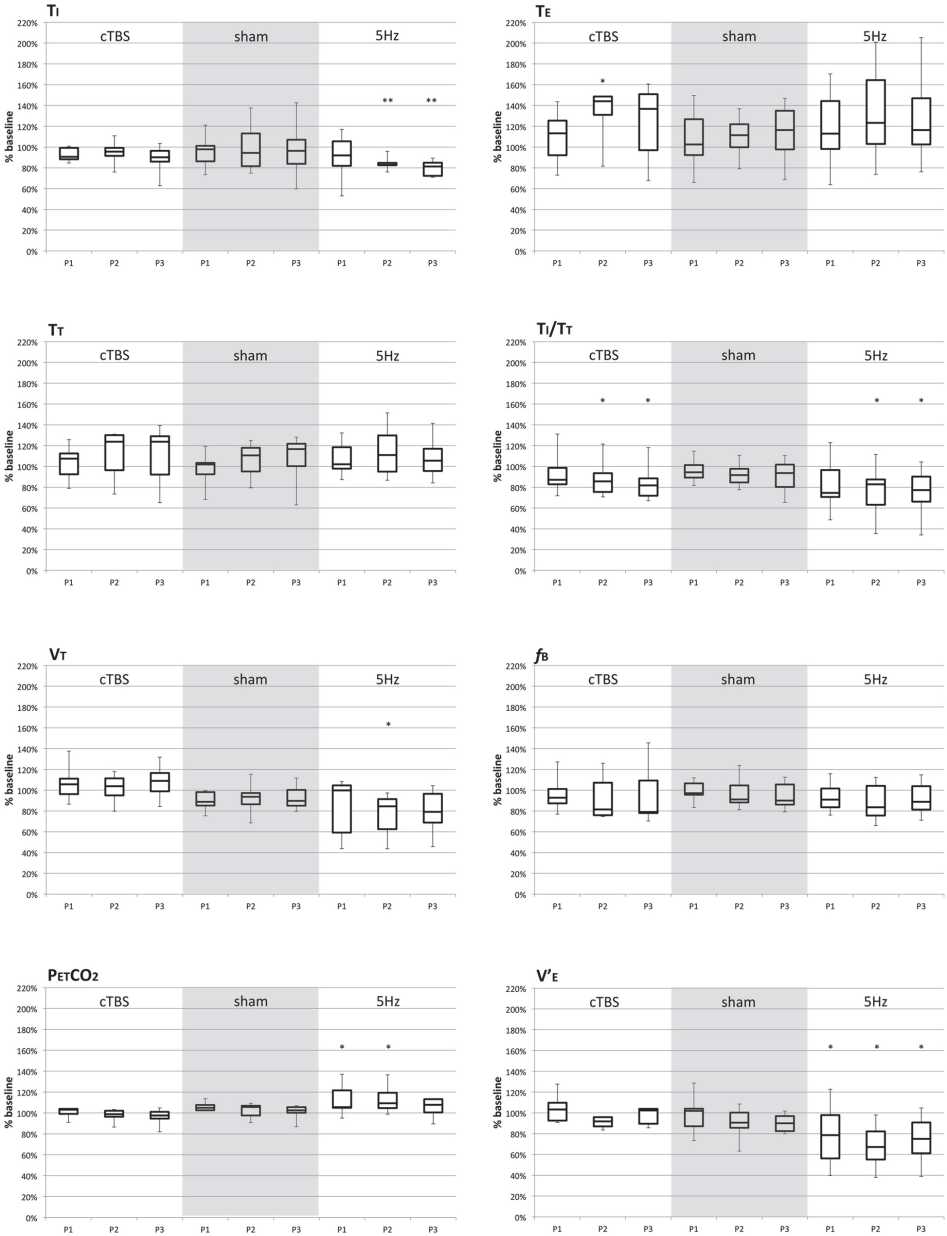
**Breathing pattern variables (expressed in percentage of baseline values) during the “POST1” period (P1), “POST 2” (P2), and “POST 3” (P3) following continuous theta-burst repetitive magnetic stimulation (cTBS; first group of three boxes), sham stimulation (grayed area, second group of three boxes), and 5 Hz continuous repetitive magnetic stimulation (third group of three boxes)**. Each box represent the interquartile range of the data, with indication of the median inside the box. The whiskers mark the minimum and maximal values of the distributions. The “^*^” denotes statistical significance at the 5% threshold. *T*_i_, inspiratory time; *T*_E_, expiratory time; *T*_T_, total cycle time; *T*_I_/*T*_T_, duty cycle; *V*_T_, tidal volume; *f*_B_, breathing frequency; *V*′_E_, ventilation; PetCO2, end-tidal expired carbon dioxide.

Following sham conditioning of the SMA, no significant changes in breathing pattern during ITL were observed (gray areas in Figure 1). In contrast, both the excitatory (5 Hz) and the inhibitory (cTBS) SMA conditioning paradigms resulted in significant breathing pattern alterations (Figure 1).

In response to the 5 Hz excitatory rTMS conditioning of the SMA, the inspiratory time (*T*_I_) and the duty cycle (*T*_I_/*T*_T_) were significantly decreased compared to BL (χ^2^ = 15.857, *p* = 0.01 for *T*_I_; *F* = 4.177, *p* = 0.021 for *T*_I_/*T*_T_). These differences were present at POST2 and POST3 (Table 1). A reduction in breathing frequency was observed, but did not reach statistical significance (*F* = 2.975, *p* = 0.059). Tidal volume was significantly less after 5 Hz conditioning (χ^2^ = 10.371, *p* = 0.016), the difference being significant at POST2 only. Overall, these changes translated into a significant reduction in ventilation (*F* = 6.82, *p* = 0.003) at POST1, POST2, and POST3 (Table 1) that was corroborated by a significant rise in PetCO2 (χ^2^ = 10.714, *p* = 0.013) at POST1 and POST2 (Table 1).

In response to inhibitory cTBS rTMS conditioning of the SMA, inspiratory time, tidal volume, breathing frequency, ventilation, and PetCO2 were unaffected (Table 2). Expiratory time (TE) was significantly lengthened (χ^2^ = 3.718, *p* = 0.031; difference present at POST2), hence a decrease in *T*_I_/*T*_T_ (*F* = 4.808, *p* = 0.012) at POST2 and POST3.

Of note, because the ITL was set in such a way as to represent a fixed percentage of maximal inspiratory pressure, some of our subjects breathed against higher absolute loads than others (4 subjects against 20–25 cm H_2_O loads, three subjects against 30–35 cm H_2_O loads). There was a trend for the rTMS-related decrease in ventilation to be more marked in the subjects facing the higher loads but this was not significant and not corroborated by PetCO2 changes (data not shown).

### Breathing discomfort

During BL, the intensity of breathing discomfort as evaluated by the VAS was 2.09 ± 0.52 cm (range: 0–4.37). There was no change after SMA conditioning (5 Hz rTMS: *F* = 1.160, *p* = 0.352; cTBS: χ^2^ = 3.6, *p* = 0.306; sham: *F* = 1.606, *p* = 0.223; Table 3).

**Table 3:** **Effects of sham stimulation, 5 Hz conditioning, and cTBS conditioning of the supplementary motor area (SMA) during inspiratory threshold loading on respiratory discomfort evaluated with VAS (cm)**.

	**Respiratory discomfort evaluated with VAS**			
	**BL**	**POST1**	**POST2**	**POST3**
SHAM	2.17 (0.69)	2.07 (0.52)	2.54 (0.71)	2.66 (0.63)
5 Hz	2.09 (0.52)	1.46 (0.32)	1.78 (0.32)	2.15 (0.50)
cTBS	1.82 [1.23–4.25]	2.09 [0.10–4.72]	1.71 [0.76–3.62]	2.08 [1.62–4.56]

BL, baseline; POST1, mean (SEM) or median [interquartile] over the first 5 min post-conditioning; POST2, over the second 5 min epoch; POST3, over the third 5 min epoch.

## Discussion

This study shows that manipulating SMA excitability with repetitive transcranial magnetic stimulation in healthy subjects can alter breathing pattern during inspiratory threshold loading and therefore disrupt inspiratory load compensation. This appears to be the first report that breathing pattern can be altered in response to non-invasive brain stimulation. The most salient finding is the sustained decrease in load-induced hyperventilation that occurred following the 5 Hz rTMS protocol. This observation challenges our hypothesis that conditioning the SMA with a known excitatory rTMS paradigm would enhance the load-induced hyperventilation, but may have implications regarding dyspnoea.

The study involved a small number of subjects. Yet, we did observe significant differences in breathing pattern, and we did so using a sham-controlled design respecting current evidence-based recommendations. Of value, the sustained reduction in V′_E_ after 5 Hz rTMS was accompanied by a concomitant and physiologically coherent rise in PetCO2: the two measures being completely independent, they corroborate one another. The changes that we observed lasted long after rTMS has ceased, and therefore cannot be explained by experimental pain or stress (which would have been expected to induced hyperventilation, not hypoventilation). We took stringent and state-of-the-art precautions to localize the SMA and strictly maintain the stimulation over it (neuronavigation with *post-hoc* check of anatomical positioning). Therefore, we are confident that the changes in breathing pattern can be interpreted as rTMS-induced respiratory plasticity. We however acknowledge that given the complexity of the descending pathways to respiratory muscles (see review in Butler et al., 2014), the effects of rTMS that we observed could result from many mechanisms.

Inhibitory conditioning of the SMA with cTBS had minimal effect (Table 2). cTBS-induced plasticity is sensitive to the history of muscle activation (Huang et al., 2008; Iezzi et al., 2008). In hand muscles, phasic voluntary movements of the target muscle after conditioning can completely reverse the effect of cTBS applied over M1 (metaplasticity; Iezzi et al., 2008). In our study, strong phasic breathing movements were present during ITL after SMA conditioning. These movements are, in part, cortically driven (Raux et al., 2007b, 2013). Therefore, it is possible that metaplasticity played a role in the absence of the expected reduction in load-induced hyperventilation following cTBS conditioning. Of note, the same cTBS protocol reduced the amplitude of diaphragmatic motor evoked potentials by ~30% in the study by Laviolette et al. (2013), but post-conditioning diaphragm motor evoked potentials were obtained during unloaded spontaneous breathing where respiratory movements are not cortically driven.

The effects of 5 Hz conditioning were strongly inhibitory (~30% reduction in ventilation at POST2; Table 1). This might suggest that the SMA exerts a baseline inhibitory action on breathing control, however this result comes in marked contrast with the excitatory nature of this protocol regarding electrophysiological outcomes (Matsunaga et al., 2005; Raux et al., 2010; Laviolette et al., 2013). Divergent impacts of rTMS on electrophysiological and motor outcomes have been described (review in Chouinard and Paus, 2010). One interpretation lies in the fact that rTMS not only modifies the excitability of the stimulated cortical area but also interferes with its cortico-cortical connections (“network effect”; Civardi et al., 2001; Matsunaga et al., 2005; Hamada et al., 2009; Lu et al., 2012). This can occur quite distantly from the stimulation site (Bestmann et al., 2004). This “network effect” may account for excitatory effects of rTMS on antagonist muscles. Of note, rTMS conditioning of the dorsal premotor cortex have dissociated effects on a given finger movement depending on its initiation (externally cued vs. self-initiated; Lu et al., 2012). Inspiratory load compensation engages not only the SMA, but a complex cortico-subcortical network comprising several areas (Raux et al., 2013) driving an intricate ensemble of inspiratory and expiratory muscles. It could be hypothesized that 5 Hz rTMS over the SMA not only modifies the excitability of the corticophrenic pathway (Raux et al., 2010; Laviolette et al., 2013) but also modifies synaptic connectivity within the above neural network, resulting in an altered load-response. Alternatively, the conjunction of ITL-induced SMA activity and of an excitatory conditioning protocol could have triggered modulating mechanisms leading to a disfacilitation of long-term plasticity and/or a facilitation of long-term depression (homeostatic plasticity, see Abraham, 2008).

The SMA has multiple roles in the planning, execution, reprogramming and inhibition of complex movements (review in Nachev et al., 2008). It also contributes to the prediction of sensory consequences of movement before movement onset (Makoshi et al., 2011) and is the source of efferent signals used by other brain areas to modulate somatosensory activity during motor actions (Haggard and Whitford, 2004). It is thought to fine-tune these actions through response evaluation (Stock et al., 2013). Motor control models posit that this efference copy contains an anticipation of the sensory consequences of movement that is compared, in a feed forward manner, to the actual afferences produced by the movement (Wolpert and Flanagan, 2001; Shadmehr et al., 2010). This comparison leads to dynamic adaptation in the event of a discrepancy. This process can be disrupted by conditioning the SMA prior to the execution of a movement (Haggard and Whitford, 2004; Makoshi et al., 2011; White et al., 2013). In this view, current theories on the pathogenesis of dyspnoea postulate that dyspnoea arises from discordance between the efference copy of ventilatory drive and the actual respiratory afferent traffic reaching the brain (Parshall et al., 2012). The ITL-induced SMA activity is associated with breathing discomfort (Raux et al., 2007a,b, 2013). Therefore, and taking into account the various limitations that have been discussed above, the SMA could belong to the brain structures contributing to the type of dyspnoea that is induced by ITL. This is all the more a reasonable hypothesis that the SMA receives afferent messages generated by inspiratory efforts (Logie et al., 1998) and could therefore be an actual efference–afference “comparison” site.

The reduction in ITL-induced hyperventilation (Table 1) after 5 Hz SMA conditioning would be expected to decrease ITL-induced breathing discomfort, but this was not the case (Table 3). This might result from too mild a load-induced discomfort in our subjects, leaving little room for improvement. The natural dispersion of the psychophysiological dyspnoea evaluation (VAS) may also have obscured a putative effect: respiratory discomfort ratings at POST1 were numerically lower than during BL after 5 Hz conditioning but not after cTBS or sham stimulation (Table 3). Of note for future research, opioids depress the cortical networks involved in volitional breathing (Pattinson et al., 2009). This could mediate, at least in part, their beneficial effects on dyspnoea. If rTMS alone proves insufficient to alleviate dyspnoea, it would be interesting to study whether and to what extent it can be used to this aim in conjunction with opioids (noting that rTMS-induced analgesia involves endogenous opioids; Taylor et al., 2012). A mere opioid sparing effect of ventilatory plasticity inducing approaches would be relevant progress in the field.

## Funding

This study was funded by a “Contrat de recherche Legs Poix de la Chancellerie de l'Université de Paris,” by the Association pour le Développement et l'Organisation de la Recherche en Pneumologie et sur le Sommeil (ADOREPS), Paris, France and by the program “Investissement d'Avenir ANR-10-AIHU 06” of the French Government. The study was also supported by a grant from the Fondation d'Entreprise Air Liquide.

### Conflict of interest statement

The authors declare that the research was conducted in the absence of any commercial or financial relationships that could be construed as a potential conflict of interest.
